# Expanding Indications for Liver Transplantation in the Treatment of Hepatocellular Carcinoma

**DOI:** 10.3390/curroncol31080355

**Published:** 2024-08-20

**Authors:** Rachel Hogen, Tara Barry, Vijay Subramanian

**Affiliations:** Transplant Institute, Tampa General Hospital, Tampa, FL 33606, USA; tarabarry@tgmg.org (T.B.); vsubramanian@tgmg.org (V.S.)

**Keywords:** hepatocellular carcinoma, liver transplantation, downstaging, local regional therapy, immunotherapy

## Abstract

Improvements in downstaging therapies have expanded the indications for liver transplantation (LT) for hepatocellular carcinoma (HCC). Patients with more advanced disease are now considered candidates due to advancements in radiation therapy, combination therapies, and immunotherapy. Combination stereotactic body radiation therapy (SBRT) and trans-arterial chemoembolization (TACE) has been shown to be superior to the historic treatment, sorafenib, in patients with macrovascular invasion. These patients are now candidates for LT with stable disease after LRT. Patients with ruptured HCC and prolonged stability have also been shown to have acceptable outcomes. The role of neoadjuvant immunotherapy needs to be further defined and has the potential to further improve tumor control prior to transplant.

## 1. Introduction

Hepatocellular carcinoma (HCC) is the most common liver cancer and a leading cause of cancer-related mortality world-wide. The main risk factors for HCC are hepatitis B and C and end-stage liver disease. Early-stage disease in patients without cirrhosis is typically treated with resection and hepatitis treatment. In patients with cirrhosis, the treatment of HCC becomes multi-disciplinary. The diseased liver is the nidus for the development of HCC, and recurrence is common. Thus, liver transplantation (LT) becomes the most definitive therapy in patients with disease confined to the liver. The Milan criteria, which define one HCC lesion less than 5 cm and three lesions less than 3 cm without macrovascular invasion, have historically been used to determine who will benefit from LT [1]. Local regional therapy (LRT) such as ablation, trans-arterial chemoembolization (TACE), and radiation therapy are used to treat and downstage HCC while patients await transplantation. Chemotherapy has limited effectiveness in HCC and has historically been reserved for patients with metastatic disease or a large tumor burden in the liver who are not candidates for other treatment modalities [2].

Advancements in local regional therapy and immunotherapy have led to an expansion in the use of LT for the treatment of HCC. Radiation therapy has improved local control in HCC, allowing for more advanced tumors to be effectively downstaged. Stereotactic body radiation therapy (SBRT) has emerged as the best treatment for intermediate-stage HCC, with better local tumor control compared to other LRT modalities. Radiotherapy alone, or in combination with TACE, has also been shown to effectively downstage tumors with macrovascular invasion. Patients with macrovascular portal branch invasion who demonstrate prolonged stability after LRT are now eligible for exception points to receive a deceased donor liver and have acceptable outcomes with living donor liver transplantation (LDLT). Similarly, case series have demonstrated successful downstaging with neoadjuvant immunotherapy. Patients with a history of ruptured HCC and prolonged stability are also now eligible for exception points for deceased donor LT.

## 2. Advances in Local Regional Therapy

Historically, LT has been indicated for patients with advanced liver disease and HCC within Milan criteria [1]. As LRT has become more effective, the indications for LT have expanded. Currently, patients can be downstaged and access deceased donor LT with priority in the United States if they are within the University of California San Francisco (UCSF) criteria (a single lesion ≤ 6.5 cm in diameter or 2 lesions ≤ 4.5 cm, with total tumor diameter ≤ 8 cm) [3]. Historically, the macrovascular invasion of the main portal vein or hepatic vein has been considered a contraindication to LT; however, Organ Procurement and Transplantation Network (OPTN) policy has recently been updated to include patients with primary portal vein branch invasion, who have remained stable for a prolonged (minimum of 12 months) interval after treatment, to be candidates for LT [4]. Thus, there is now a role for downstaging patients with primary portal branch invasion and advanced interventional techniques to achieve this.

Patients with portal venous thrombosis (PVT) and larger tumors can be more challenging to treat. Radiofrequency ablation is the standard treatment option for small, early-stage tumors less than 2 cm that are not amenable to surgical resection. Trans-arterial chemoembolization (TACE) is the standard treatment option for intermediate-stage lesions; however, patients with HCC and portal vein thrombosis (PVT) are not amenable to TACE due to the high risk of ischemia and liver failure [5]. Trans-arterial radioembolization (TARE) is an option in intermediate-stage HCC with PVT. Furthermore, while TARE is associated with a more significant side-effect profile, and randomized controlled studies demonstrate some mixed results, it has been shown to be superior to TACE in Barcelona Clinic Liver Cancer (BCLC) stage A and B [6,7]. A recent randomized controlled trial demonstrated a significantly better survival rate (median overall survival 30.2 months after TARE versus 15.6 months after TACE, hazard ratio (HR), 0.48; *p* = 0.006) and likelihood of downstaging to transplant (nine downstaged to transplant with TARE compared to four with TACE) with TARE compared to TACE in patients with intermediate-stage (Barcelona Clinic Liver Cancer stage A and B) HCC [7]. However, TARE has yet to be shown to be superior to sorafenib in patients with intermediate-stage HCC and PVT [8,9].

### Stereotactic Body Radiation Therapy

Radiotherapy has now become the most effective form of tumor control for intermediate-stage HCC. Radiation therapy can now be given with more precision and reduced side effects to surrounding liver tissue. Furthermore, radiation therapy is not limited by tumor location and can be given in the setting of portal vein or macrovascular invasion. The most common type of radiation therapy is photon therapy or external beam radiation therapy. Several delivery systems are clinically available, including conventional fractionated radiotherapy (CFRT), hypo-fractionated RT (HFRT), particle radiotherapy (PRT) and stereotactic body radiation therapy (SBRT). Stereotactic body radiation therapy is effective for the treatment of all stages of HCC and is the optimal therapy for intermediate-stage HCC, and it allows for more prolonged tumor control while patients await LT [10]. Stereotactic body radiation therapy is an effective alternative to ablation therapy for early-stage, small HCC, with increased efficacy compared to ablation as the tumor size increases. Local control rates in this population have been reported between 70 and 90% at 2 years [11,12,13,14,15,16]. In fact, a large systemic review and meta-analysis of propensity score studies by Eriguchi et al. demonstrated comparable overall survival and significantly better local control with SBRT compared to RFA [16]. A randomized trial comparing proton beam therapy (PBT) to RFA for patients with recurrent HCC with a tumor size less than 3 cm and less than or equal to two total tumors, demonstrated that PBT was non-inferior to RFA [17].

Stereotactic body radiation therapy has been increasingly shown to improve local tumor control without significant differences in overall survival compared with TACE. Stereotactic body radiation therapy may have more limited applications to Child–Pugh class B patients and larger tumors due to toxicity [10]. There is now randomized controlled trial data comparing TACE and SBRT. The TRENDY Trial by Moelker et al. compared time to progression in noncirrhotic or Child’s Class A cirrhotic patients with one to three tumors up to a cumulative diameter of less than 6 cm who were ineligible for surgery or ablation between TACE with drug-eluding beads (DEBs) and SBRT. Median time to progression (12 months for TACE with DEB and 19 months for SBRT; *p* = 0.15) and overall survival (36.8 months for TACE with DEB and 44.1 months for SBRT; *p* = 0.36) were equivalent between the two groups [18]. However, post hoc analysis demonstrated 100% local control rates at 1 and 2 years for SBRT compared to 54.4% and 43.6% local control rates at 1 and 2 years for TACE with DEB (*p* = 0.019). There was no toxicity reported in the SBRT group, while 13% of the patients in the TACE-DEB arm were reported to suffer ≥ grade 3 treatment toxicity [18]. Scorsetti et al. published a single-center, prospective, randomized controlled trial comparing SBRT versus TACE after an incomplete response following TACE in Child’s Class A or B cirrhotic patients with BCLC stage A or B HCC. Their primary endpoint was local control [19]. Significantly, better local control was demonstrated with SBRT vs. TACE (median not reached vs. 8 months; *p* = 0.0002), corresponding to 1-year local control rates of 84% and 23% for SBRT and TACE, respectively [19]. Overall survival and incidence of distance recurrences were equivalent between the two groups. There were no grade >3 toxicities in either group [19]. Bush et al. published a prospective randomized controlled trial of subjects with untreated HCC meeting Milan or UCSF transplant criteria comparing PBT or TACE. Patients with Child’s Class C cirrhosis or bilirubin greater than 3 were excluded. They similarly found no difference in overall survival; however, progression-free survival and local control were significantly better with PBT [20]. Several propensity-score-matched analyses have also demonstrated improved local control rates at 2 years with SBRT compared to TACE (78.2% vs. 23% and 91% vs. 67.2%, respectively) [21,22].

## 3. Hepatocellular Carcinoma with Portal Branch Invasion

With advancements in LRTs, patients with HCC with macrovascular portal branch invasion are now candidates for transplantation if they demonstrate prolonged stability after treatment. Historically, patients with HCC and macrovascular invasion have had poor prognosis, with median survival between 2 and 6 months, and not been candidates for LT [23,24,25,26]. Sorafenib has been the primary treatment modality for this patient population, despite it only prolonging survival by an average of 2–3 months [2]. However, macrovascular invasion has been shown to, in some cases, have complete response to local radiation therapy, yielding prolonged survival in palliative patients, with 1-year overall survival reported at 44.9% in patients treated with SBRT and 60.9% in patients treated with particle radiotherapy in pooled analysis [27].

Radiation therapy in combination with TACE has yielded the best results in treating HCC with macrovascular portal invasion. Trans-arterial chemoembolization and TARE have not demonstrated improved survival or local control compared to sorafenib [28]. Furthermore, TACE in the setting of PVT can significantly increase the risk of liver ischemia because there is no collateral blood supply to the treatment area [28]. However, radiation therapy works synergistically with TACE. Radiation therapy appears to target vascular invasion and recanalize the portal vein to allow entry of the chemoembolization products. Trans-arterial chemoembolization in combination with radiotherapy has demonstrated significantly better local tumor control and overall survival than sorafenib in patients with HCC and portal branch invasion [22,29]. In a single-center, randomized trial of patients with HCC with macrovascular invasion confined to the liver, TACE combined with radiation therapy compared to sorafenib alone demonstrated improved 12-week, progression-free survival (86.7% vs. 34.3%, *p* < 0.001), a significantly better radiologic response (33.3% vs. 2.2%; *p* < 0.001), a significantly longer time to progression (31 vs. 11.7 weeks; *p* < 0.001), and a significantly longer overall survival (55 vs. 43 weeks; *p* = 0.04) [29]. Hepatocellular carcinoma with macrovascular portal invasion has 70% pooled response rates to SBRT [30]. Particle radiotherapy appears to be the most promising radiation therapy as it can more precisely target tumors, limiting toxicities to surrounding tissues [27].

Radiotherapy is an effective bridge to transplantation in patients with favorable tumor biology and HCC with portal invasion (Table 1). While outcomes in this population are inferior to those of other patients with HCC within Milan or UCSF criteria, they are far superior to prolonged sorafenib [25,31,32]. While the UNOS now has a pathway to exception points for these patients, living donor liver transplantation (LDLT) offers a chance at long-term survival that can be timed with LRT for those that have donors. Several studies in the living donor population have demonstrated success at using radiotherapy as a bridge to LT in patients with HCC and macrovascular portal branch invasion [31,32,33,34,35]. Soin et al. evaluated the outcomes of 25 patients with portal vein tumor thrombosis (PVTT) who underwent downstaging followed by LDLT. All but one patient had grade VpI-Vp3 PVTT (tumor thrombus located in second-order, beyond second-order, or first-order branches). One patient had tumor thrombus in the main portal vein [32]. Their downstaging protocol included SBRT with or without TACE or TARE. Response evaluation was undertaken at 4–6 weeks, and then LDLT was performed if successful downstaging was achieved. Successful downstaging was defined as the loss of 18-FDG avidity on PET scan and/or the disappearance of PVTT enhancement on contrast CT. Forty-three patients had HCC within UCSF criteria and PVTT (*n* = 38) or hepatic vein or inferior vena cava thrombosis (*n* = 5) [32]. Sixty-three percent (27 patients) were successfully downstaged. Twenty-five of the successfully downstaged patients underwent LDLT. One-, three-, and five-year OS in downstaged patients who underwent LDLT was 75%, 53%, and 53%. One-, three-, and five-year recurrence-free survival was 78%, 78%, and 52% [32]. Serenari et al. conduced a prospective study of 17 patients with HCC and PVTT who underwent downstaging with TARE, followed by DDLT in those with a complete radiologic response after 6 months. Five patients (29.4%) were successfully downstaged and underwent transplant. The five-year overall survival was 60% compared to 0% of patients who did not make it to transplant (*p* = 0.03). There was, however, a 60% recurrence rate within 1 year [36]. Choi et al. evaluated the outcomes of LDLT in patients with PVTT. Five-year DFS and OS were 14.3% in patients with lobar or Vp3 branches. However, patients with segmental PVTT had better outcomes with 5-year DFS and OS rates of 63.9% and 50.3% [31]. However, the use of LRT was not specified in the study by Choi et al. [31].

Overall, the outcomes of LT for HCC with PVTT are inferior to most other indications for LT. Thus, patient selection is paramount. Tumor biology is likely important in the outcomes of LT for HCC with PVTT, just as it is in HCC overall. Complete or partial response of the PVTT to radiotherapy or LRT is an important prognostic indicator [32]. Lobar PVTT, as opposed to segmental PVTT, also portends a worse prognosis [32]. Furthermore, in patients with favorable tumor biology, PVTT should be considered a locoregional disease rather than a systemic disease. Thus, low alpha-fetoprotein, low FDG-18 PET avidity, and small tumor sizes are important prognostic indicators in this group of patients. In addition, a waiting period to demonstrate stable disease before pursing transplant is also important [25].

Liver transplantation after a prolonged stability post-HCC rupture is another recently expanded indication for liver transplantation in the treatment of HCC. Historically, ruptured HCC was a contraindication to LT because it is considered a T4 disease. However, in 2023, the OPTN updated its policy to make patients with a history of ruptured HCC and no evidence of metastatic disease after 12 month eligible for exception points [4]. This policy is supported largely by case reports; however, it demonstrates the heterogeneity in HCC tumor biology and the importance again of patient selection [39,40,41,42]. Lee et al. looked retrospectively within a single center at patients who underwent LDLT after ruptured HCC. Of the five patients identified, four (80%) survived greater than 5 years. Two experienced a recurrence within the follow-up duration of 85 months—one initially developed a peri-hepatic metastatic lesion, and the second, a rectal serosal mass from peritoneal seeding. Both were initially managed with surgical resection; one survived 12.4 months post transplant, and the second survived 79.1 months post transplant [43].

## 4. Immunotherapy for Hepatocellular Carinoma

There is growing evidence for the use of immunotherapy as a form of downstaging therapy [44,45]. Historically, tyrosine kinase inhibitors (TKIs) (sorafenib and lenvatinib) and anti-vascular endothelial growth-factor monoconal antibodies (bevacizumab) have been the first-line therapies available for the treatment of HCC [2,46]. The benefits of these systemic therapies were limited; sorafenib improved median survival by 3 months [23,46].

Just like with other cancers, immune checkpoint inhibitors have shown promise in the treatment of HCC. One of the earliest trials was the IMBrave050 trial, which showed improved survival by over 6 months for patients with atezolizumab + bevacizumab compared to sorafenib [47]. This result was also demonstrated in the HIMALAYA trial, with improved survival for patients on duravalumab plus tremelimumab [48].

Early evidence for the use of immunotherapy in HCC is in the adjuvant setting. For example, the patient selection for immunotherapy is based on those with a high risk for HCC recurrence, after either resection or local ablative procedures [47,48]. This includes features such as tumor size >5 cm or total number of tumors and burden. There are more trials in the pipeline that are awaiting results. Overall, these seem promising, considering the best previous therapy available for these patients was sorafenib, which demonstrated a limited survival benefit [2].

## 5. Neoadjuvant Therapy for Hepatocellular Carcinoma

As a natural extension to the positive results from immunotherapy in the adjuvant setting, there is increasing interest in its use as neoadjuvant therapy. Immunotherapy in the neoadjuvant setting is considered to be favorable due to the presence of active tumor cells that will facilitate immune activation with better antigen recognition and a higher likelihood of activation of pathways to mediate tumor cell killing. This is particularly useful for clinically non-detectable micro-metastatic disease. These primed antitumor cells may also remain active after other curative modalities that may further prevent the recurrence of a tumor [45].

Among the various studies showing the use of neoadjuvant therapy for HCC, there have been upwards of 70% necrosis in tumors that were resected. A PD-1 blockade alone (nivolumab or cemiplimab) or in combination with TKI (cabozatinib) or CTLA4 (ipilmumab) have shown a radiological response rate of around 25%, with a pathological response rate up to 33%. These results are from a single-center experience, and large-scale trial results are still awaited [49]. Nevertheless, the responses from these studies, including in patients with “advanced” disease, have provided some evidence that they may be advantageous in downstaging therapy to patients with HCC outside the Milan criteria.

## 6. Immunotherapy and Liver Transplantation

There are several case series reported with the use of immunotherapy in LT [50,51,52,53,54,55]. As stated before, immunotherapy primes the immune system, and there may be a persistent memory effect. Early studies with nivolumab in organ transplant recipients showed approximately 50% rejection (including in those who received LT). In general, immunotherapy post LT is contraindicated due to high rates of rejection and graft loss [51]. The first case of the use of pre-LT immunotherapy resulted in massive hepatic necrosis and patient loss soon after transplant [56]. This profound effect and risk of rejection appears to be related to the timing of immunotherapy. Studies with patients receiving PD-1 blockade up to 4 weeks prior to LT showed that this can be used safely with lower rejection rates and some modifications in immunosuppression [50,54]. These results come in a limited number of patients and mostly from single-center case series [51,52,53,54,55]. Immune-related events post transplant has been seen in patients up to 1 year post transplant, and hence, keeping this in consideration is important. Effects directly related to immunotherapy have been noted in around 35% of patients in the literature [50,52,54].

In the absence of randomized trials, no conclusions or recommendations can be made for immunotherapy for downstaging or neoadjuvant therapy for HCC prior to transplant. However, there is certainly some promise that, either by itself or in combination with other locoregional therapy in patients with non-metastatic disease, there may be some benefit [57]. The most recent European Society of Transplantation (ESOT) consensus statement refrains from making any recommendations in both the use of immunotherapy pre-transplant and the best way to measure response to therapy [58]. The Organ Procurement and Transplantation Network (OPTN) guidelines now recommend that patients who have received pre-transplant immunotherapy for HCC should be evaluated on a case-by-case basis to see if they are eligible for MELD exception points for HCC [4]. This is certainly a step in the right direction.

The decision on when to hold immunotherapy is still under debate. It is recommended to stop immunotherapy at least 8–12 weeks (2–3 half-life) prior to liver transplantation [51]. This is feasible for patients with planned LDLT; however, it may not be feasible for those awaiting deceased donor LT. The other question that remains unanswered is whether one therapy is better than the other (in terms of TKI/PD-1/CTLA-4). As the overall experience with these drugs increases and randomized trials are designed to answer the role on immunotherapy in the pre-transplant setting, we will hopefully have better guidelines.

## 7. Conclusions

Liver transplantation is now an option for patients with more advanced HCC due to advancements in LRT. Combined SBRT and TACE have demonstrated far superior outcomes to sorafenib in patients with macrovascular invasion. Now patients with macrovascular invasion and stability after LRT can be offered LT. Immunotherapy has emerged as an additional, promising, downstaging tool that warrants further larger studies.

## Figures and Tables

**Table 1:** Liver transplantation after downstaging for macrovascular invasion.

Study	Year	# of Patients	LRT	RFS	OS
Soin et al. [32] LDLT after downstaging for PVTT	2020	25	SBRT in combination with TACE, TARE, or ablation	5 year—51%	5 year—57%
Jeong et al. [33] LDLT after downstaging for major vascular invasion	2017	17	Radiotherapy combined with TACE	1 year—70.6% 3 year—57.8%	1 year—87.4% 3 year—60.5%
Serenari et al. [36] DDLT after downstaging for PVTT	2021	5	TARE	1 year—40%	5 year—60%
Choi et al. [31] LDLT after downstaging for PVTT	2017	5	TACE and CRT	1 year—60%	1 year—100% 2 year—80%
Levi et al. [37] DDLT after downstaging for macrovascular invasion	2017	4	TARE	39.1 months	39.1 months
Han et al. [38] LDLT after downstaging with chemoradiotherapy and HAIC	2016	8	Chemoradiotherapy HAIC	1 year—87.5%	Median—33 months

Living donor liver transplantation (LDLT); portal vein tumor thrombus (PVTT); deceased donor liver transplantation (DDLT); local regional therapy (LRT); recurrence-free survival (RFS); overall survival (OS); stereotactic body radiation therapy (SBRT); trans-arterial chemoembolization (TACE); trans-arterial radioembolization (TARE); conformational radiotherapy (CRT); hepatic arterial infusion chemotherapy (HAIC).
